# Assessment of the first presentations of common variable immunodeficiency in a large cohort of patients

**DOI:** 10.1186/s12865-023-00545-4

**Published:** 2023-06-13

**Authors:** Hossein Esmaeilzadeh, Armita Jokar-Derisi, Amir Hossein Hassani, Reza Yazdani, Samaneh Delavari, Hassan Abolhassani, Negar Mortazavi, Aida Askarisarvestani

**Affiliations:** 1 Division of Allergy and Clinical Immunology, Department of Pediatrics, School of Medicine, Shiraz University of Medical Sciences, Shiraz, Iran; 2 Allergy Research Center, Shiraz University of Medical Sciences, Shiraz, Iran; 3 Student Research Committee, School of Medicine, Shiraz University of Medical Sciences, Shiraz, Iran; 4 School of Medicine, Shiraz University of Medical Sciences, Shiraz, Iran; 5 Research Center for Immunodeficiencies, Children’s Medical Center, Tehran University of Medical Sciences, Tehran, Iran; 6 Primary Immunodeficiency Diseases Network (PIDNet), Universal Scientific Education and Research Network (USERN), Tehran, Iran; 7 Department of Neurology, Thomas Jefferson University, Philadelphia, PA USA; 8 Department of Biosciences and Nutrition, Karolinska Institutet, Stockholm, Sweden; 9 Department of Clinical Pharmacy, School of Pharmacy, Shiraz University of Medical Sciences, Shiraz, Iran; 10 Department of Pediatrics, Namazee hospital, Shiraz University of Medical Sciences, Shiraz, Iran

**Keywords:** Common variable immunodeficiency, Primary immunodeficiency, Clinical immunology

## Abstract

**Background:**

Common Variable Immunodeficiency (CVID) is a primary immunodeficiency syndrome resulting in recurrent infections, autoimmunity, and granulomatous manifestations.

**Methods and materials:**

This retrospective study was conducted on an Iranian national registry of immunodeficient patients from 2010 to 2021. The frequency of first presentations of CVID and its association with sex, age of onset, and family history of CVID was evaluated.

**Results:**

A total of 383 patients entered the study, 164 of whom were female, and the rest were male. The mean age of the patients was 25.3 ± 14.5 years. The most frequent first presentations of CVID were pneumonia (36.8%) and diarrhea (19.1%). Patient sex, age of onset, and family history did not make significant differences in first presentations of this disease.

**Conclusion:**

pneumonia is the most common first presentation of CVID. Family history of CVID, the age of symptom onset, and sex made no differences in the first presentations of CVID.

## Highlights

Pneumonia is the most common first presentation of CVID.

Female patients developed CVID at a younger age than male patients.

Sex is not a differentiating factor in first presentations of CVID.

Family history does not change the first presentations of CVID.

Adulthood-onset and childhood-onset CVID were not different regarding the first presentations.

## Introduction

Common Variable Immune Deficiency (CVID) is a spectrum of signs and symptoms indicating Primary Immune Deficiency (PID). CVID patients mostly experience defective antibody production, antibody class switch recombination, or antibody affinity maturation [1]. Patients afflicted with this condition may suffer from a wide range of symptoms that will end in about half of them being deceased in a 12.5-year period [2]. The highest proportion of patients with PID (40.2%) in the United States are diagnosed with CVID [3]. In Western Europe, CVID has caused 10,510 years of life lost due to death and disability for each 100,000 of the general population [4].

The presentations of CVID can be divided into two major classes: infectious and non-infectious. Recurrent infections in CVID vary from pneumonia to upper respiratory tract infections and gastrointestinal involvement as well as osteomyelitis and septic arthritis with bacterial pathogens being the leading etiologies of the infectious complications [5, 6]. Non-infectious manifestations of CVID can be in form of autoimmunities such as autoimmune hemolytic anemia, autoimmune thrombocytopenia, autoimmune thyroiditis, and inflammatory bowel disease [7]. Granulomatous disease and malignancies are also seen with a higher prevalence in CVID patients compared to the general population [8].

No previous study has assessed the first presentations of this disease and the role of first symptoms in the evaluation of the prognosis; consequently, in this study, we aimed to collect the first presentations of CVID in a large cohort of the Iranian National Registry of Primary Immune Deficiencies.

## Methods and materials

This retrospective study is a part of a large cohort study on Iranian immunodeficiency patients’ national registry from early 2010 to late 2021 [9]. All the patients with a confirmed diagnosis of CVID were included in this study. For any patient labeled as a case of CVID, the following data were collected: date of birth, age, sex, age of onset (month), age of diagnosis (month), having a positive family history of immunodeficiency, delayed diagnosis, and the first presentation. CVID diagnosis was made by the use of European Society of Immunodeficiency criteria as follows: significantly low levels of IgG as well as either IgM or IgA or both with no isohemagglutinins and/or poor response to inoculated vaccines in patients older than two years and in whom other causes of hypogammaglobulinemia have been ruled out [10].

The data were entered into IBM SPSS version 26.0. In this descriptive study, values are presented as numbers (percentage) for qualitative variables and as mean ± standard deviation (SD) for quantitative variables. Comparison of the clinical data and presentations were made using an independent two-sample T-test for quantitative variables and a chi-square test for qualitative variables. P-values less than 0.05 were considered statistically significant.

This study was approved by the Shiraz University of Medical Sciences Committee for Ethics in Biomedical Research by the code IR.SUMS.MED.REC.1400.24398 and written consent forms were obtained from the patients or their legal guardians prior to the interview.

## Results

In our study, 383 patients with CVID were surveyed. The patients aged 1–69 years with a mean age of 25.3 ± 14.5 years. Among the studied patients, 164 (42.8%) were female and 219 (57.2%) were male. The median age at onset was 2.0 years (IQR 0.5–8.7) and a mean of 6.9 ± 10.8 years. The median age at diagnosis was 10.0 years (IQR 3.1–21.0) with a mean of 14.4 ± 13.9 years. The median time of delay in diagnosis was 4.0 years (IQR 1.0-9.6) and a mean of 7.6 ± 9.7 years. The mean follow-up period was 9.7 ± 8.6 years in our study. Forty-seven patients (12.3%) had a positive family history of CVID and 193 patients (50.4%) had parental consanguinity.

To evaluate the patients for their diagnosis of CVID immunologic tests were checked the results of which are summarized in Table 1. In our study, the patients had a total White Blood Cell (WBC) count of 10310.2 ± 14210.00 /mm^3^. The mean IgG level was measured to be 308.6 ± 339.38 mg/dL which is diminished for the adult population. The mean IgM and IgA levels were 44.2 ± 65.24 and 30.8 ± 55.03 mg/dL.

**Table 1:** Immunologic tests of the participants

parameter	mean	SD
WBC (N = 362)	10310.2	14210.00
Lymphocyte count (N = 345)	3554.6	3987.9
Neutrophil count (N = 340)	5283.0	7795.07
CD3 count (N = 298)	2516.7	2870.3
CD4 count (N = 302)	1150.0	1307.4
CD8 count (N = 293)	1304.5	1741.0
CD56 count (N = 63)	277.8	450.4
CD19 count (N = 287)	498.3	
CD20 count (N = 108)	495.1	950.71
IgG (N = 363)	308.6	339.38
IgG1 (N = 13)	216.2	208.53
IgG2 (N = 16)	96.4	182.46
IgG3 (N = 18)	63.6	90.10
IgG4 (N = 17)	7.3	13.80
IgA (N = 353)	30.8	55.03
IgM (N = 362)	44.2	65.24
IgE (N = 250)	28.4	138.39
Isohemagglutinin A (N = 77)	1.5	7.25
Isohemagglutinin B (N = 67)	4.3	21.35
Anti-tetanoid (N = 82)	0.6	1.28

In our study, 356 patients reported their first presentations. The list of first reported presentations of CVID is summarized in Table 2. In total, 484 presentations were reported as the first presentations by our patients. Pneumonia was the most frequent first reported presentation of CVID (36.3%). Diarrhea (18.8%), otitis (14.4%), sinusitis (12.1%), other upper respiratory tract infections (7.2%), skin lesions (5.4%) and fever (3.6) were the other frequent first reported presentations of CVID by the patients.

**Table 2:** Frequencies of the first reported presentations in 383 studied patients with Common variable immunodeficiency

Presentation	n (%)
Pneumonia	141 (36.8)
Diarrhea	73 (19.1)
Otitis	56 (14.4)
Sinusitis	47 (12.3)
Upper respiratory infection	28 (7.3)
Skin lesion	21 (5.5)
Fever	14 (3.6)
Lymphadenopathy	8 (2.1)
Autoimmunity	8 (2.1)
Failure to thrive	7 (1.8)
Arthritis	7 (1.8)
Oral candidiasis	6 (1.5)
Meningitis	5 (1.3)
Allergic asthma	5 (1.3)
Jaundice	5 (1.3)
Bloody diarrhea	4 (1.0)
Urinary tract infection	4 (1.0)
IBD	1 (0.26)
lymphoma	1 (0.26)
Other	43 (11.2)

Other presentations: Anemia, Anorexia, Bronchiectasis, Carcinosis, Chickenpox, Conjunctivitis, Eczema, Diabetes Mellitus, Fatigue, Lung tumor, Malnutrition, Measles, Neutropenia, Hypocalcemia, Osteomyelitis, Parotitis, Nausea, weight loss

Male and female patients were compared regarding their first presentation of CVID, the results of which are summarized in Table 3. The mean age at onset was significantly higher in male patients compared to females (8.4 versus 5.9 years respectively, P-value = 0.045). the mean of age, age at diagnosis, and time of delay in diagnosis were similar in both male and female patients (P-values > 0.05). Also, the family history of CVID in male and female patients was similar (P-value = 0.290).

**Table 3:** Comparison of patient characteristics between male and female CVID patients

	Male (n, 219)	Female (n, 164)	P-value
Age (year)	25.6 ± 13.9	25.1 ± 14.9	*0.748
Age at onset (year)	8.4 ± 12.8	5.9 ± 8.8	*0.045
Age at diagnosis (year)	14.9 ± 14.6	14.1 ± 13.4	*0.596
Delay in diagnosis (year)	6.8 ± 8.9	8.2 ± 10.2	*0.213
Follow-up period (year)	9.4 ± 7.9	10.0 ± 9.1	*0.549
Family history of CVID	22 (16.2)	24 (21.4)	†0.290

Data are mean ± SD or number (%)

P-values calculated using *Independent Sample t-test or †Chi Square test

**Fig. 1:**
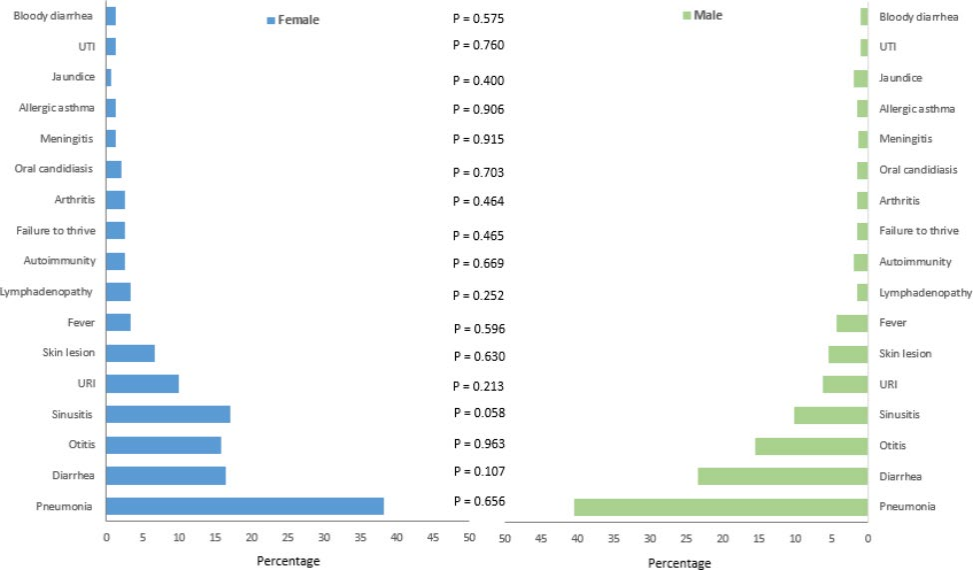
Differences in the frequency of the first reported presentations in 256 studied male and female patients with Common variable immunodeficiency. Differences between male and female patients were not statistically significant (P-values > 0.05)

Figure 1 shows the differences in the frequency of the first reported presentations in studied male and female CVID patients. Pneumonia was the most common first reported presentation in both male and female patients. In the female patients, diarrhea and otitis were the other common first reported presentations respectively, whereas, in the male patients, sinusitis and diarrhea were in the second and third rank, respectively. Differences between male and female patients were not statistically significant (P-values > 0.05).

The patients were also compared regarding their initial symptoms based on the age at which the first symptoms developed. The information regarding the age at the time of symptom onset was available for 337 patients, of who 294 (87.2%) experienced childhood-onset CVID and 43 patients (11.1%) experienced adulthood-onset CVID. The patients were divided into two groups: adulthood-onset (first symptoms after 18 years old) and childhood-onset (first symptoms presented before 18 years old). The symptoms were found to have no association with the age of onset. Table 4 summarizes the findings of this analysis.

**Table 4:** Comparison of common CVID presentation in regards to patients age at onset

	Age at onset		
	< 18 year (N = 294)	≥ 18 year (N = 43)	P-value
Pneumonia	112 (38.1%)	20 (46.5%)	0.233
Diarrhea	60 (20.4%)	9 (20.9%)	0.878
Otitis	45 (15.3%)	4 (9.3%)	0.320
Sinusitis	36 (12.2%)	8 (18.6%)	0.221
Upper respiratory infection	21 (7.1%)	4 (9.3%)	0.526
Skin lesion	17 (5.8%)	1 (2.3%)	0.711
Fever	14 (4.8%)	0 (0.0%)	0.231
Autoimmune hematologic disease	6 (2.0%)	2 (4.7%)	0.263
Asthma/allergy	5 (1.7%)	0 (0.0%)	0.511

Data are number (%)

P-values calculated using Chi Square test or Fisher’s Exact Test

Finally, the patients were compared regarding their first presentations based on the family history of CVID. The information regarding the family history of CVID was available for 249 patients, 202(81.12%) of who had a negative family history of CVID and 47 (18.88%) who had a positive family history of CVID. No association was found between family history of CVID and the first presentations of CVID. Table 5 summarizes the findings of the comparison.

**Table 5:** Comparison of common CVID presentation in regards to family history of CVID

	Family history of CVID		
	No (N = 202)	Yes (N = 47)	P-value
Pneumonia	69 (35.8)	18 (41.9)	0.453
Diarrhea	46 (23.8)	5 (11.6)	0.079
Otitis	32 (16.6)	5 (11.6)	0.419
Sinusitis	31 (16.1)	9 (20.9)	0.442
Upper respiratory infection	18 (9.4)	2 (4.7)	0.544
Skin lesion	14 (7.3)	3 (7.0)	0.949
Fever	11 (5.7)	2 (4.3)	0.700
Autoimmune hematologic disease	5 (2.5%)	1 (0.5%)	0.555
Asthma or allergy	3 (1.5%)	1 (0.5%)	0.700

Data are number (%)

P-values calculated using Chi Square test

In this study, 58 patients underwent endoscopy/colonoscopy during the course of their disease. The most frequent finding was lymphocytic nodular hyperplasia and/or aggregations of lymphocytes (37.9%) in the gastrointestinal tract wall, followed by villous atrophy (26.2%). Moreover, fifty-eight patients had a lung computed tomography (CT) scan in whom the most frequently found abnormality was bronchiectasis (53.4%) followed by peribronchial thickening (29.3%). Pulmonary function test was performed for 59 patients which were normal in 47.5% of the cases. Furthermore, 20.3% of the cases had an obstructive pattern in their pulmonary function test. The findings of these investigations are summarized in Table 6.

**Table 6:** Findings of endoscopy/colonoscopy, lung CT scan, and pulmonary function test in CVID patients

investigation	finding	frequency	Percentage
Endoscopy/colonoscopy	Esophagitis	14	24.1%
	Gastritis	19	32.8%
	duodenitis	16	27.6%
	Enteritis	8	13.8%
	colitis	20	33.9%
	Lymphocytic nodular hyperplasia	22	37.9%
	Villous atrophy	21	26.2%
	malabsorption	7	12.1%
	Gastroesophageal reflux disease	5	8.6%
Lung CT scan	Normal	2	3.4%
	emphysema	26	44.8%
	bronchiectasis	31	53.4%
	Fibrotic changes	7	12.1%
	Atelectasis/segmental collapse	13	22.4%
	pleuresis	2	3.4%
	calcification	2	3.4%
	Peribronchial thickening	17	29.3%
	Mass/lymphadenopathy	7	12.1%
	nodule	6	10.3%
	Pleural effusion	1	1.7%
	consolidation	13	22.4%
Pulmonary function test	normal	28	47.5%
	obstructive	12	20.3%
	restrictive	11	18.6%
	Mixed pattern	8	13.6%

In this study, 199 patients had whole exome sequencing, 20.2% of whom had no definitive mutations for CVID. The most frequently seen pathogenic mutation was in LRBA (16.8%), followed by DNMT3B (8.4%). Two of the patients had double mutations. Table 7 summarizes the mutations in this cohort of patients.

**Table 7:** Pathogenic mutations found in whole exome sequencing of patients with CVID

gene	frequency	Percentage
nothing	24	20.2%
AIRE	2	1.7%
BAFFR	2	1.7%
BLNK	2	1.7%
BRAC2	1	0.8%
BTK	3	2.5%
CD27	2	1.7%
CD70	1	0.8%
DCK1	1	0.8%
DCLRE1C	1	0.8%
DNM-TBB	1	0.8%
DNMT3B	10	8.4%
FOXP3	1	0.8%
ICOS	1	0.8%
IKZF1	1	0.8%
IL12B	1	0.8%
IL12RB1	2	1.7%
IL21R	1	0.8%
ITCH	1	0.8%
JAK3	1	0.8%
LRBA	20	16.8%
NEHJ1	1	0.8%
NFKB1	2	1.7%
NFKB2	1	0.8%
NKFB1	1	0.8%
PGM3	1	0.8%
PHC1	1	0.8%
PIK3CD	3	2.5%
PIK3R1	3	2.5%
PMS2	3	2.5%
RAC2	3	2.5%
RAG1	3	2.5%
RFX5	1	0.8%
RFXANK	3	2.5%
SH2DA1	1	0.8%
STAT2	1	0.8%
STAT3	2	1.7%
TACI	4	3.4%
MAGT1	1	0.8%
TERT	1	0.8%
XIAP	2	1.7%
ZBTB24	4	3.4%
µ heavy	1	0.8%

## Discussion

In this article, we evaluated the first presentations of CVID in all of the newly diagnosed patients in Iran in a ten-year period. We found that respiratory infections, diarrhea, and otitis were the most common first presentations of CVID in our studied population.

In our study, we found that female patients were diagnosed earlier than male patients; however, this difference was not statistically significant. This finding might root in the difference in health-seeking behaviors of male and female patients. In a study by Khajeh et al., they found that male patients were more likely to practice self-medication instead of referring to their doctor. They were also more inattentive to their healthcare needs [11].

According to Ochs, CVID signs and symptoms can be categorized into three groups; first, symptoms associated with infections and immune deficiency, second, symptoms correlated to autoimmunity and granuloma formation, and third, manifestations of the frequent malignancies that patients with CVID are predisposed to such as lymphoma and leukemia [12]. In our article, all of the proposed manifestations were taken into consideration when the patients were approached.

In a systematic review conducted by Zainaldain et al., they found that pneumonia was the most frequently seen infection in patients with CVID with an overall prevalence of over two-thirds. Upper respiratory tract infections and gastrointestinal tract involvement with infectious pathogens were the next common infective complications of CVID [5]. In our article, pneumonia was the most common first presentation of CVID involving about 36.3% of the patients, followed by diarrhea (18.8%), otitis (14.4%), and sinusitis (12.1%). The findings of our study are in line with previous studies regarding the order of frequent first presentations. However, the prevalence of such findings is vastly different which might root in the different methodologies used in various studies. Moreover, in our study, the studied population is rather generalizable since it is conducted on a large population of a national registry.

In a study by Janssen et al., they systematically reviewed the pooled prevalence of various CVID presentations. They found that pneumonia was present in about 75% of the patients, while we found that pneumonia was responsible for about one-third of the first presentations [6]. This difference might indicate that CVID can predispose patients to the development of pneumonia in later stages. Such mechanisms might be through the immune deficiency, and other lung involvements such as chronic pulmonary disease, bronchiectasis, and interstitial lung disease among others. They also found that the “infection-only” type of CVID was less prevalent in adults and the adults showed more interstitial lung disease and structural abnormalities compared to children which are in line with the explanation.

In a study by Baloh et al., the authors assessed patients with CVID over 30 years. They found that children were less likely to develop malignancies and more likely to develop autoimmune hematologic diseases. However, in our study, no association was found between the age of symptom onset and any symptoms. This difference might root in the fact that longer follow-up in the study of Baloh made it possible to detect the long-term consequences of CVID, however, in our study such long follow-up was not possible [13].

In a study by Westh et al. on the Danish population, they found that the median age at onset was 29 years, however, the mean age at onset was 8.4 years for males and 5.9 years for females in our study [14]. This difference might root in the variety of healthcare systems in the two countries of Denmark and Iran. in Iran, strict guidelines are less abided by and the coverage of insurance companies is not dependent on the physician’s function, therefore, more expensive tests are performed sooner compared to developed countries such as Denmark. This fact might eliminate the recall bias in the interview with the patients. In the study performed in Denmark men were diagnosed earlier in contrast to our study. Consequently, the age at diagnosis was lower in Iran and the delay in diagnosis was shorter in Iran compared to Denmark. Another explanation of these differences might be the multifactorial nature of CVID in which genetics and environment both play important roles in the pathogenesis of the disease.

In a study by Gathmann et al., they assessed CVID patients in European countries and found that male patients had a younger age of onset which was not the case in our study [15]. They postulated that the earlier onset of the symptoms in male patients might be due to an undiagnosed X-linked primary immunodeficiency. However, in our study, such immunodeficiency syndromes were ruled out.

In a study by Hosseini-Chavoshi et al. they found that more than one-third of marriages in Iran were consanguineous, while in our study about half of the patients had consanguineous parents which shows the importance of inheritability in this disease [16].

We also found that no association was found between the family history of CVID and the first presentations of CVID. However, no previous study was found in this regard.

## Conclusion

In conclusion, we found that pneumonia, diarrhea, otitis, and sinusitis were the most frequent first presentations of CVID. Female patients showed the symptoms earlier than the male patients and there was no statistically significant difference between male and female patients regarding the first presentation of CVID. Family history of CVID and age of onset made no difference in the first presentations of CVID.

## Data Availability

The data belongs to the Iranian national registry of primary immunodeficiency and the data used for this study can be available from corresponding author upon reasonable request.

## Abbreviations

CVID: Common Variable Immunodeficiency
PID: Primary Immunodeficiency
IBD: Inflammatory Bowel Disease
WBC: White Blood Cell
SD: Standard Deviation
CT: Computed Tomography
